# Stigma of Dementia During COVID-19: First Insights From a Twitter Study

**DOI:** 10.1093/geroni/igab046.3442

**Published:** 2021-12-17

**Authors:** Juanita-Dawne Bacsu, Megan O'Connell, Alison Chasteen, Sarah Fraser on behalf of CCNA's SSI Working Group

**Affiliations:** 1 University of Saskatchewan, Saskatoon, Saskatchewan, Canada; 2 University of Toronto, Toronto, Ontario, Canada; 3 University of Ottawa, Ottawa, Ontario, Canada

## Abstract

Stigma is a critical issue that reduces the quality of life for people living with dementia and their care partners. Despite this knowledge, little research examines stigma of dementia, especially within the context of the COVID-19 pandemic. This presentation aims to: 1) identify the contributing factors of stigma against dementia during the COVID-19 pandemic; and 2) describe actions to challenge stigma of dementia. Using Twitter data, tweets were compiled with Python’s GetOldTweets application from February to September 2020. Search terms included keywords for dementia (e.g., Alzheimer’s) and COVID-19 (e.g., coronavirus). From the 20,800 tweets, filters were used to exclude irrelevant tweets. The remaining 5,063 tweets were analyzed by a group of coders with 1,743 tweets identified for further stigma-related coding. The 1,743 tweets were exported to Excel for thematic analysis and divided among 13 coders. Each tweet was coded independently by two reviewers to ensure intercoder reliability (e.g., 86%). Contributing factors of stigma of dementia included: ageism and devaluing the lives of people with dementia (e.g., ‘old and dying anyways’); misinformation and false beliefs (e.g., ‘COVID-19 vaccine causes dementia’); political dementia-related slander and ridicule (e.g., ‘dementia Joe’); and stigma within healthcare and long-term care organizations (e.g., pushing DNR orders). Globally, there is an urgent need for more dementia education and awareness targeted towards the general public, healthcare workers, and policymakers to reduce stigma against people living with dementia. Further research is necessary to explore the contributing factors and interventions to reduce stigma of dementia during the COVID-19 pandemic and beyond.

